# Coronary Artery Magnetic Resonance Angiography Combined with Computed Tomography Angiography in Diagnosis of Coronary Heart Disease by Reconstruction Algorithm

**DOI:** 10.1155/2022/8628668

**Published:** 2022-03-23

**Authors:** Yun Ling, Jiapei Qiu, Jun Liu

**Affiliations:** Department of Cardiovascular Surgery, Ruijin Hospital, Shanghai Jiaotong University School of Medicine, Shanghai 200025, China

## Abstract

This research aimed at discussing the diagnosis effect of coronary artery magnetic resonance angiography (MRA) combined with computed tomography (CT) angiography (CTA) based on the back-projection filter reconstruction (BPFR) algorithm in coronary heart disease (CHD), and its role in the diagnosis of coronary artery disease (CAD). Sixty patients with CHD were selected and randomly rolled into group A (undergone MRA examination), group B (undergone CTA examination), and group C (undergone MRA + CTA), with 20 cases in each group. Taking the diagnostic results of coronary angiography as the gold standard, the MRA and CTA images were reconstructed using a BPFR algorithm, and a filter function was added to solve the problem of image sharpness. In addition, the iterative reconstruction algorithm and the Fourier transform analysis method were introduced. As a result, the image clarity and resolution obtained by the BPFR algorithm were better than those obtained by the Fourier transform analytical method and the iterative reconstruction algorithm. The accuracy of group C for the diagnosis of mild coronary stenosis, moderate stenosis, and severe stenosis was 94.02%, 96.13%, and 98.01%, respectively, which was significantly higher than that of group B (87.5%, 90.2%, and 88.4%) and group C (83.4%, 89.1%, and 91.5%) (*P* < 0.05). The sensitivity and specificity for the diagnosis of noncalcified plaque in group C were 87.9% and 89.2%, respectively, and the sensitivity and specificity for the diagnosis of calcified plaque were 84.5% and 78.4%, respectively, which were significantly higher than those in groups B and C (*P* < 0.05). In summary, the BPFR algorithm had good denoising and artifact removal effects on coronary MRA and CTA images. The combined detection of reconstructed MRA and CTA images had a high diagnostic value for CHD.

## 1. Introduction

With the increasingly serious urban aging, more and more people suffer from cardiovascular disease (CVD) [1]. A recent epidemiological survey indicated that there are currently 290 million patients with CVD in China, of which more than 10 million are with coronary heart disease (CHD) and 4.5 million are patients with heart failure. The number of deaths from CVD accounts for the highest number of deaths from all diseases, far higher than other diseases such as tumors. For every 10 deaths, 4 patients die from CVD [2]. The most common CVD is CHD. The treatment cost of CHD patients has brought a huge burden to the family and society, and CHD has attracted more and more scholars' attention [3]. Early diagnosis and early prevention of CHD patients can effectively reduce the medical expenses of patients, while also avoiding the waste of medical resources. At present, the commonly used diagnostic methods for CHD include electrocardiogram (ECG) (to preliminary judge the location of myocardial ischemia or infarction), chest X-ray (to exclude lung disease, preliminary assessment of patients with suspected heart failure), myocardial markers (to determine the myocardial injury status and predict the time of myocardial ischemia of patients), computed tomography (CT) angiography (CTA), and magnetic resonance angiography (MRA) [4].

Stress echocardiography can determine whether the patient has signs of myocardial ischemia during exercise, so as to further clarify the diagnosis of stable CHD. CTA is a noninvasive inspection method to determine the degree of coronary artery stenosis (CAS) and its branches. If there is no stenosis on the coronary CT angiography, the invasive inspection is generally not necessary [5, 6]. Magnetic resonance angiography (MRA) is an examination method that uses electromagnetic waves to generate images of two-dimensional or three-dimensional structures of the body, and uses magnetic resonance phenomena to obtain electromagnetic signals from the human body and reconstruct human information [7]. It can be used for the diagnosis of heart disease, cardiomyopathy, pericardial effusion, mural thrombosis, etc. For the diagnosis of CHD, branches of coronary artery lesions can be intuitively found, which is of great significance for assessing the degree of branch obstruction and judging the severity of disease [8, 9]. Image reconstruction technology plays an important role in many fields, and there are a series of extremely complex image processing and mathematical calculation problems in the research and implementation of the reconstruction algorithm [10, 11]. The essence of back-projection reconstruction is to evenly erase (back projection) the ray projection taken from the finite object space onto all points in the infinite space where the ray reaches, including the point with the original pixel value of 0 [12, 13]. At present, algorithms such as image reconstruction and computer-assisted medical image analysis have obvious advantages in major breakthroughs in technology and improvement of medical level, and have also become an effective way to solve medical image problems [14, 15].

At present, CTA and MRA are widely used in the screening of CHD. However, there are few studies on the combination of the two in the diagnosis of CHD. In addition, the two diagnostic methods mentioned above have certain limitations, such as high false-positive rate and large amount of contrast agent. The resolution of coronary artery CTA is limited, and serious artifacts may exist during reconstruction, leading to inaccurate coronary artery assessment. Therefore, an attempt was made to construct a back-projection filter reconstruction (BPFR) algorithm, which convolved the Ramp-Lak filter with sin(*x*)/*x* to obtain the Sheep–Logan filter. The MRA images and noisy data of coronary artery were reconstructed by using the Sheep–Logan filter, so as to study the diagnostic value of coronary artery MRA and CTA combined detection based on the reconstruction algorithm for CHD.

## 2. Research Objects and Major Methods

### 2.1. General Data of Research Objects

In this study, there are 60 patients with CHD in hospital from November 2019 to October 2020, 38 males and 22 females, with an average age of 43.21 ± 8.37 years. The patients were randomly divided into group A (undergone MRA examination), group B (undergone CTA examination), and group C (undergone MRA + CTA), with 20 in each group. The study had been approved by the ethics committee of hospital, and the patients had been informed about this study and singed informed consents.

Inclusion criteria: patients without a history of CVD-related surgery; those with age between 50 and 70 years; and those taking no other drugs and antibiotics.

Exclusion criteria: patients with other system or organ diseases; those with cardiomyopathy; those who were allergic to iodine contrast agents; and those with serum creatinine above normal levels.

### 2.2. CTA and MRA Examinations

For patients in group A (undergone MRA examination), the coronary artery MRA used 1.5 T superconducting MR machine, the gradient field strength was 40 mT/m, the switching rate was 150 T/m per second, an 8-channel heart coil was adopted, and the heart electrical trigger and respiratory monitoring device were equipped. For the breath-hold three-dimensional (3D) fast steady-state balance precession sequence, the time of repetition (TR) was 4.1 ms, the time of echo (TE) was 1.9 ms, and the reversal angle was 65. The field of view (FOV) was 26 × 26 cm, the matrix was 256 × 192, partial K-space sampling was adopted, the layer thickness was 3.0 cm with 8–10 layers, and ECG-triggered mid-diastolic end-expiratory sampling was adopted. The maximum intensity projection coronary artery reconstruction method was used to reconstruct the coronary artery image.

For patients in group B (coronary artery CTA examination), the 16-slice spiral CT was used for CTA. The scanning range was determined by phase scanning, the upper tracheal carina was below the level, the lower boundary was 1 cm below the diaphragmatic surface, and the left and right sides were 1∼2 cm larger than both sides of the heart edge. Layer selection was made by observing the left pulmonary trunk plane located on the calcification integral image. Scanning sequence: heart rate 50∼60 beats/min was prospectively gated, and heart rate 60∼70 beats/min was retrospectively gated. Maximum density projection recombination was used.

For patients in group C, coronary MRA and coronary CTA combined examination was performed. The diagnostic accuracy of coronary angiography was evaluated by the gold standard.

### 2.3. Image Reconstruction

**Figure 1:**
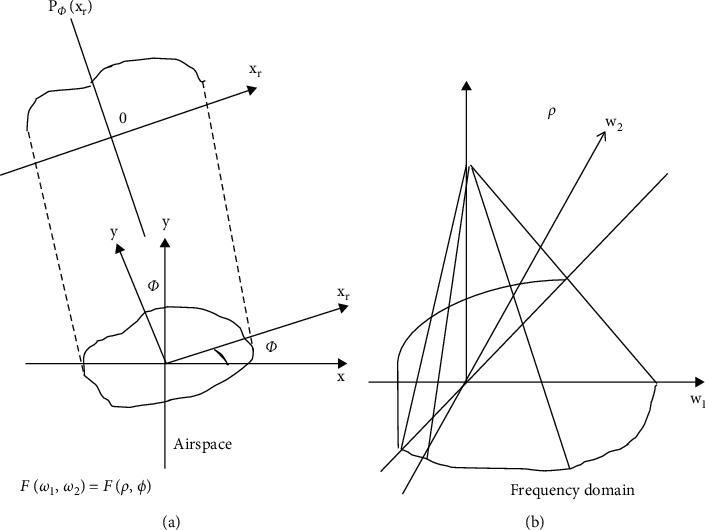
Principle of image reconstruction. (a) Before FT and (b) one-dimensional (1D) and two-dimensional (2D) FT.

Image reconstruction algorithms are roughly classified into Fourier transform (FT) algorithms and iterative reconstruction (IR) algorithms. With the increasing application of computer technology, there are various reconstruction algorithms with different characteristics. The BPFR algorithm, which is a FT theory-based spatial processing technology, was adopted as the basic algorithm of the model. It can perform convolution on the projection of each acquisition projection angle before back projection, thereby enhancing the quality of the reconstructed image. The reconstruction principle is shown in Figure 1.

In the process of the Fourier slice theorem, the one-dimensional FT of the projection and the 2D FT of the original image were equivalent. The Fourier slice theorem can perform the FT on the projections to obtain a 2D FT from a projection [16, 17]. Therefore, the projection image can be reconstructed by collecting enough projections at different times (180 acquisitions generally), solving the 1D FT of each projection, combining abovementioned slices into the 2D FT of the image, and then using the inverse FT [18].

The BPFR algorithm back-projects the measured projection data along the scanning path “original path” to the pixels passed by the path. The value of a pixel in the tomographic plane is regarded as the accumulation (or average) of all the projection values of the rays passing through the pixel [19, 20]. The direct back-projection method is simple and easy to implement, but the image of Deba is very fuzzy, and it needs more time-consuming follow-up correction to restore the original image. In the parallel beam scanning mode, in addition to the fixed coordinate system *x-y* and the polar coordinate system (*γ*, *ϕ*), a rotating coordinate system *t-s* was adopted for easy principal explanation here. The coordinate system *t-s* coincided with the origin of the coordinate system *x-y*, and the angle was *θ*. Therefore, the ray position can be uniquely determined by the coordinate (*t*, *θ*), and (*t*, *θ*) corresponded to a ray projection value. The ray (*t*, *θ*) can pass (*γ*, *ϕ*) by satisfying thefollowing equation as follows:

(1) $$\begin{array}{c} t=r\cos\left(\phi -\theta \right). \end{array}$$

In the translation/rotation scan mode, the scan operation is stepped by angular increment Δ and step distance *d*, so (*t*, *θ*) is often expressed as a discrete quantity (*n*  *d*, *m*Δ), and *m* and *n* are integers. Corresponding projection data is given as follows:

(2) $$\begin{array}{c} p\left(t,\theta \right)\left(t=nd,\theta =m\Delta \right). \end{array}$$

*t*=*nd*, *θ*=*m*Δ was also discrete. △ and *d* must be small enough; otherwise, those rays passing (*γ*, *ϕ*) will not pass the actual discrete ray position (*nd*, *m*Δ):

(3) $$\begin{array}{c} f\left(\gamma ,\phi \right)=\frac{1}{m}\sum_{m=0}^{M-1}\tilde{p}m\Delta \left[r\cos\left(\phi -m\Delta \right)\right]. \end{array}$$

In the above equation,

(4) $$\begin{array}{c} r\cos\left(\phi -m\Delta \right)\neq nd. \end{array}$$

Therefore, the following equation cannot be directly obtained, and interpolation must be performed:

(5) $$\begin{array}{c} \tilde{p}m\Delta \left[r\cos\left(\phi -m\Delta \right)\right], \end{array}$$

(6) $$\begin{array}{c} t=nd, \end{array}$$

(7) $$\begin{array}{c} \theta =m\Delta . \end{array}$$

The above two equations were both discrete. For a certain point (*x*_*i*_, *y*_*j*_) in space, there must be a ray *t*_*m*_ under a certain angle of view *θ*=*θ*_*m*_=*m*Δ, which was defined as

(8) $$\begin{array}{c} t_{m}=x_{i}\cos\theta_{m}+y_{j}\sin\theta_{m}. \end{array}$$

Since (*x*_*i*_, *y*_*j*_) was the pixel coordinate of any point in space, *t*_*m*_ was not exactly an integer multiple of *d* and may be between *n*_0_*d* and (*n*_0_+1)*d*, that is,

(9) $$\begin{array}{c} t_{m}=\left(n_{0}+\delta \right)d,0<\delta <1. \end{array}$$

After linear interpolation, the following equation is obtained:

(10) $$\begin{array}{c} \tilde{p}\left(t_{m},\theta_{m}\right)=\tilde{p}m\Delta \left[\left(n_{0}+\delta \right)d\right],\\ \tilde{p}m\Delta \left[\left(n_{0}+\delta \right)d\right]=\tilde{p}m\Delta \left(n_{0}d\right)+\frac{\tilde{p}m\Delta \left[\left(n_{0}+1\right)d\right]-\tilde{p}m\Delta \left[\left(n_{0}d\right)\right]}{d}\left(t_{m}-n_{0}d\right). \end{array}$$

If *d* = *l*, and the fixed angle of view △ was omitted, then the below equation could be obtained:

(11) $$\begin{array}{c} \tilde{p}\left(n_{0}+\delta \right)=\tilde{p}\left(n_{0}\right)+\delta \left[\tilde{p}\left(n_{0}+1\right)-\tilde{p}\left(n_{0}\right)\right]=\left(1-\delta \right)\tilde{p}\left(n_{0}\right)+\tilde{\delta p}\left(n_{0}+1\right). \end{array}$$

Therefore, it needed to calculate *n*_0_ and *δ* first. The image area is usually divided into NXN pixels. The beam rotates around the center of the image area after translation. For any pixel (*x*_*i*_, *y*_*j*_) and viewing angle *θ*, the following equations can be satisfied:

(12) $$\begin{array}{c} t=x_{i}\cos\theta +y_{j}\sin\theta ,\\ x_{i}\cos\theta +y_{j}\sin\theta =\left(i-\frac{N}{2}\right)\cos\theta +\left(j-\frac{N}{2}\right)\sin\theta ,\\ \left(i-\frac{N}{2}\right)\cos\theta +\left(j-\frac{N}{2}\right)\sin\theta =\left(i-1\right)\cos\theta +\left(j-1\right)\sin\theta -\frac{N}{2}\left(\cos\theta +\sin\theta \right). \end{array}$$

After interpolation, the passed ray projection value can be obtained, as shown in the following equation:

(13) $$\begin{array}{c} \tilde{p}\left(t_{m},\theta_{m}\right)=\tilde{p}m\Delta \left[\left(n_{0}+\delta \right)d\right]. \end{array}$$

After it was incorporated into *f*(*γ*, *ϕ*), the following could be acquired:

(14) $$\begin{array}{c} f\left(\gamma ,\phi \right)=\frac{1}{m}\sum_{m=0}^{M-1}\tilde{p}\left(t_{m},\theta_{m}\right). \end{array}$$

In concrete realization, it can be expressed as follows:

(15) $$\begin{array}{c} f\left(i,j\right)=\sum_{m=0}^{M-1}\tilde{p}\left({\tilde{t}}_{m}\left(i,j\right),m\Delta \right). \end{array}$$

When the above equation is executed on a computer, it can be calculated as follows:

(16) $$\begin{array}{c} f_{m}\left(i,j\right)=f_{m-1}\left(i,j\right)+\tilde{p}\left({\tilde{t}}_{m}\left(i,j\right),m\Delta \right),\;m=1,\;2,\;3,\;\ldots ,\;M. \end{array}$$

### 2.4. Image Reconstruction Results

**Figure 2:**
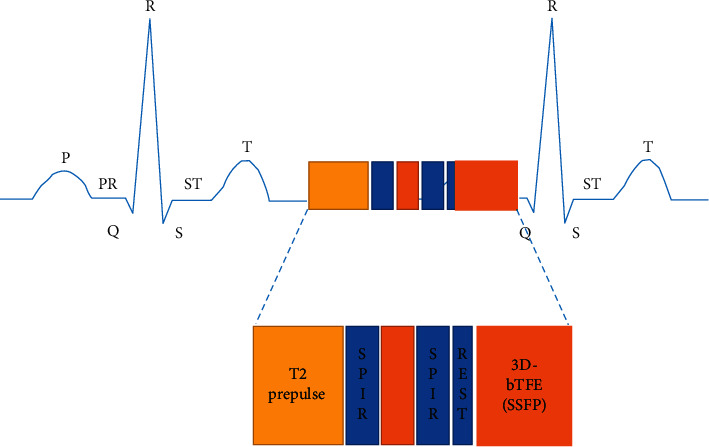
Image acquisition.

A BPFR algorithm was used to reconstruct three-dimensional coronary MRA and CTA images of patients with CHD, and a filter function was added to solve the problem of image sharpness. The image acquisition process is shown in Figure 2. Among them, P, Q, R, S, and T represented the P wave, Q wave, R wave, S wave, and T wave in the ECG, respectively. PR and ST represented the P-R interval and ST segment, respectively, in the ECG. In addition, an iterative reconstruction algorithm (reconstructing the image by solving a system of linear equations) and an analytical method of Fourier transform were introduced. For patients with different degrees of coronary stenosis, coronary calcified plaque, and noncalcified plaque, the reconstructed three-dimensional images were used to present the lesions more clearly in a three-dimensional and visualized form to achieve the effect of simulation.

### 2.5. Statistical Methods

The data were analyzed and processed with SPSS 19.0. The measurement data and count data were expressed by the mean ± standard deviation (®*x* ± *s*) and percentage (%), respectively. Pairwise comparison was realized by the analysis of variance. The difference was statistically significant at *P* < 0.05.

## 3. Results

### 3.1. Running Time of Different Algorithms

**Figure 3:**
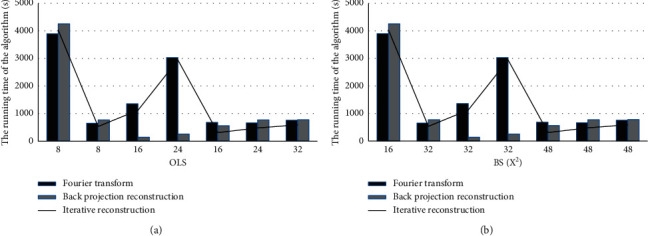
Running time for image reconstruction of different algorithms. (a) Running time at different OLSs and (b) running time at different BSs.

As given in Figure 3, the running time of the FT method and the IR algorithm was the shortest both when the overlap step (OLS) was 8 and the block size (BS) was 32^2^ and when the OLS was 16 and the BS was 482. When the OLS was 16 and the BS was 32^2^, the running time of the BPFR algorithm was the shortest.

### 3.2. Coronary CTA and MRA

**Figure 4:**
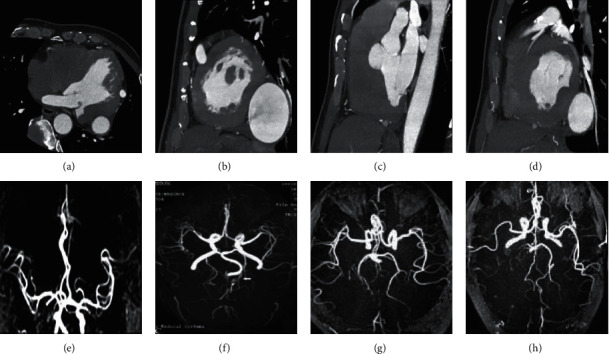
Coronary angiography images. (a–d) CTA images of four patients; (e–h) coronary MRA images of four patients.

After the R wave trigger delay of the ECG, the image acquisition was started, then coronary MRA angiography was started, then T2 prescan (inhibits venous and myocardial signals) was carried out, and then spectral presaturation inversion to restore fat saturation (inhibits fat signals) was performed, and navigation pulses were used for breathing exercises compensate.  Figure 4 shows coronary CTA and MRA images in patients with CHD. Whole-heart coronary MRA images showed severe stenosis and occlusion of the left main coronary artery and proximal anterior descending artery. There was also significant stenosis in the posterior descending artery, and the results of coronary CTA and MRA were in good agreement.

### 3.3. Reconstruction of Coronary Angiography Image

**Figure 5:**
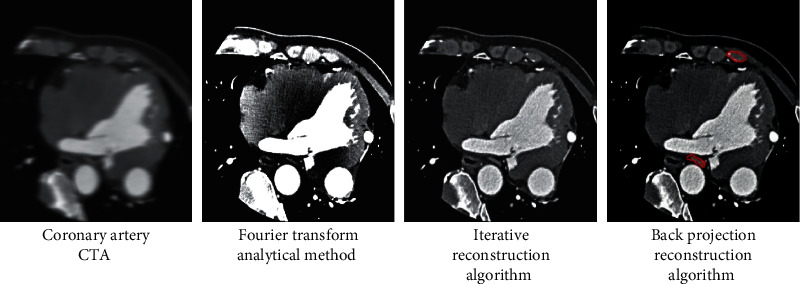
Coronary CTA image reconstruction. (a) Coronary CTA image; (b) Fourier transform analysis method reconstructed image; (c) iterative algorithm reconstructed image; (d) BPFR-reconstructed image. The red area in the figure represents coronary stenosis and plaque.

The BPFR algorithm applied the filter function to the original data, filtered signals in the image, removed the information that causes noise and artifacts, and reconstructed the image to remove the noise and artifacts of the image. Figure 5 shows the CTA images of CHD patients and reconstruction results of the FT method, IR algorithm, and BPFR algorithm. The clarity and resolution of the CTA image reconstructed by the BPFR algorithm were better than those of the FT method and IR algorithm, and the recognition rate of CAS and plaque was higher.

**Figure 6:**
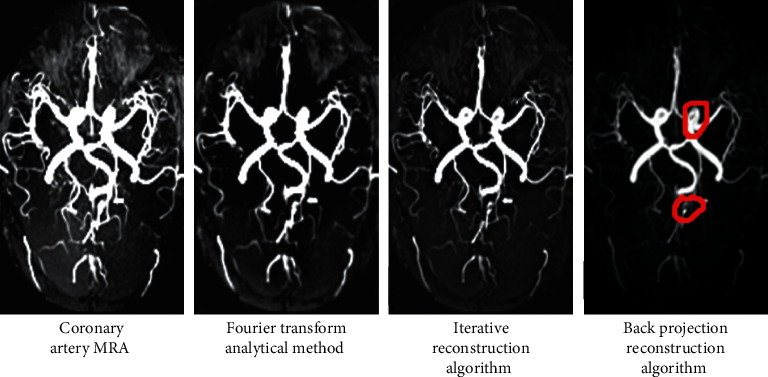
Reconstruction of the coronary artery MRA image. (a) Coronary artery MRA image; (b–d) images reconstructed by the FT method, IR algorithm, and BPFR algorithm, respectively.

BPFR corrected the structural information of the target image block through the adaptive selection of images and super-resolution reconstruction, so as to obtain high-resolution images with higher definition, clear image edges, and significantly enhanced details. Then, the interference was eliminated to reconstruct a clearer image.  Figure 6 shows the coronary artery MRA images of CHD patients and the reconstruction results of the FT method, IR algorithm, and BPFR algorithm. The red markers in the figure indicate severe stenosis and occlusion of LMCA and PADA branches. The clarity and resolution of the CTA image reconstructed by the BPFR algorithm are better compared with the FT method and IR algorithm, and the recognition rate of CAS and plaque was higher.

### 3.4. The Diagnosis Results of Coronary Artery Plaque

**Figure 7:**
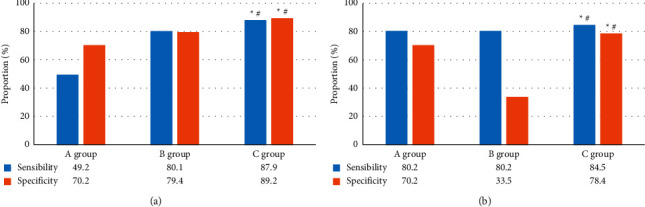
The diagnosis results of coronary artery plaque. (a, b) diagnosis results of noncalcified plaque and calcified plaque, respectively. ^*∗*^ and ^#^ indicate *P* < 0.05 vs the group A and group B, respectively.

Figure 7 shows the comparison of the sensitivity and specificity of coronary artery noncalcified plaque and calcified plaque using coronary MRA examination, coronary CTA examination, and MRA combined with CTA examination in three groups of patients. The sensitivity and specificity of the noncalcified plaque diagnosis of group C patients were 87.9% and 89.2%, respectively, which were obviously higher in contrast to the other two groups (*P* < 0.05). Sensitivity and specificity of the calcified plaque diagnosis in group C were 84.5% and 78.4%, respectively, which were higher dramatically than the values in the other two groups (*P* < 0.05).

### 3.5. The Diagnosis Results of CAS

**Figure 8:**
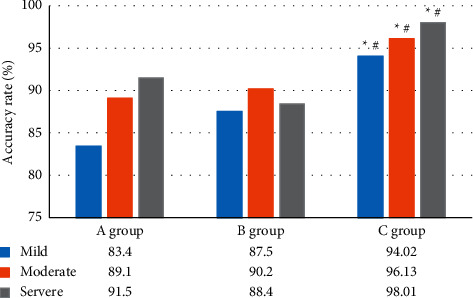
The diagnosis results of CAS. ^*∗*^ and ^#^ indicate *P* < 0.05 compared with the group A and group B, respectively.

Figure 8 illustrates the accuracy comparison results of patients in the diagnosis of mild, moderate, and severe CAS. The diagnosis accuracy of patients in group C for mild stenosis, moderate stenosis, and severe stenosis of the coronary arteries was 94.02%, 96.13%, and 98.01%, which were dramatically higher than those in group B (87.5%, 90.2%, and 88.4%) and group C (83.4%, 89.1%, and 91.5%), showing statistically obvious differences (*P* < 0.05).

## 4. Discussion

Coronary MRA is a new method of coronary artery imaging without ionizing radiation and noninvasive, which is expected to replace the current coronary angiography technology. It is currently the most used method to obtain free-breathing 3D MRA by using respiration and ECG gating technology [21]. Shen et al. [22] found that MRA can detect significant CAD and predict serious cardiac adverse events. In addition, MRA has a long imaging time and low spatial resolution. However, with the application of new technologies such as high-field magnetic resonance imaging and multichannel cardiac coils, MRA can be imaged in a relatively short period of time to accurately diagnose coronary artery disease. The BPFR algorithm is developed based on back projection, solving the problem of image sharpness by adding filter function. Without the addition of filtering function, the reconstructed image is fuzzy, while the reconstructed image with the addition of filtering function can make it clearer [23]. Roh et al. [24] included patients with stable chest pain and compared them with standard treatment and coronary artery CTA. At 4.8 years of follow-up, the incidence of death from CHD or nonfatal myocardial infarction (2.3%) was significantly lower in patients treated with coronary CTA combination therapy. Coronary artery CTA may be used to determine the event risk of nonblocking CHD, which has a positive application value for the prevention and diagnosis of CHD. In this study, MRA and CTA images of coronary artery in patients with CHD were reconstructed by reverse projection image reconstruction algorithm, and filtering function was added to solve the problem of image sharpness. In addition, the iterative reconstruction algorithm and Fourier transform analytic method were introduced.

After the BPFR algorithm was employed to process MRA and CTA images, it was found that when the OLS was 16 and the BS is 32^2^, the running time of the BPFR algorithm was the shortest. Therefore, BS was set to 32^2^ and OLS length was set to 16 pixels to obtain a high-quality image and to prevent the parameters setting from affecting the reconstruction result. The sensitivity and specificity of patients in group C for diagnosing noncalcified plaques were 87.9% and 89.2%, respectively, and those for the diagnosis of calcified plaques were 84.5% and 78.4%, respectively, which were greatly higher than the values in other groups (*P* < 0.05). It may be because CTA is more conducive to judging the degree of stenosis of coronary arteries and their branches, while MRA is more conducive to identifying the diseased branches of coronary arteries and assessing the degree of branch obstruction. Both have high specificity, so their combined use can effectively improve the diagnostic effect. The accuracy of the combined detection of patients in group C for the diagnosis of mild, moderate, and severe CAS was 94.02%, 96.13%, and 98.01%, respectively, which were much higher (*P* < 0.05). Such findings show similarity to the results of Puz et al. [25], which may be due to the noninvasive inspection method of CTA, which is suitable for the degree of stenosis of coronary arteries and their branches. MRA can obtain electromagnetic signals, which is suitable for identifying the branch of coronary artery disease, assessing the degree of branch obstruction, and judging the severity of the disease. Therefore, the combination and application of MRA and CTA testing may be more conducive to improving the diagnosis of CHD.

## 5. Conclusion

In this study, the BPFR algorithm was constructed and applied to the MRA and CTA images of coronary artery patients with CHD, and the image was reconstructed to remove noise and artifacts. It was attempted to obtain the clear image and identify the lesion, so as to study the diagnostic value of coronary artery MRA and CTA combined detection based on the reconstruction algorithm. The results showed that the BPFR algorithm had good denoising and artifact removal effects on coronary MRA and CTA images. The combined detection of reconstructed MRA and CTA images has a high diagnostic value for CHD. However, the sample size included in this study is small and lacks certain representativeness. In addition, there is no in-depth study on whether the combined application of the two detection methods will cause certain side effects to patients. Therefore, this aspect will be improved and optimized in the follow-up study, and the application of coronary artery MRA and CTA combined detection based on the reconstruction algorithm in the diagnosis of CHD will be further analyzed. In conclusion, this study provides a reference for the application of intelligent algorithms in medical imaging and disease diagnosis.

## Data Availability

The data used to support the findings of this study are available from the corresponding author upon request.
